# The ethics of relationality in implementation and evaluation research in global health: reflections from the Dream-A-World program in Kingston, Jamaica

**DOI:** 10.1186/s12910-018-0282-5

**Published:** 2018-06-15

**Authors:** Nicole A. D’souza, Jaswant Guzder, Frederick Hickling, Danielle Groleau

**Affiliations:** 1 Department of Psychiatry, Division of Social and Transcultural Psychiatry, McGill University, Montreal, Canada; 2 CARIMENSA (Caribbean Institute of Mental Health and Substance Abuse), University of the West Indies, Kingston, Jamaica

**Keywords:** Ethics, Relationality, Children, Implementation and evaluation, Global health research

## Abstract

**Background:**

Despite recent developments aimed at creating international guidelines for ethical global health research, critical disconnections remain between how global health research is conducted in the field and the institutional ethics frameworks intended to guide research practice.

**Discussion:**

In this paper we attempt to map out the ethical tensions likely to arise in global health fieldwork as researchers negotiate the challenges of balancing ethics committees’ rules and bureaucracies with actual fieldwork processes in local contexts. Drawing from our research experiences with an implementation and evaluation project in Jamaica, we argue that ethical research is produced through negotiated spaces and reflexivity practices that are centred on relationships between researchers and study participants and which critically examine issues of positionality and power that emerge at multiple levels. In doing so, we position ethical research practice in global health as a dialectical movement between the spoken and unspoken, or, more generally, between operationalized rules and the embodied relational understanding of persons.

**Summary:**

Global health research ethics should be premised not upon passive accordance with existing guidelines on ethical conduct, but on tactile modes of knowing that rely upon being engaged with, and responsive to, research participants. Rather than focusing on the operationalization of ethical practice through forms and procedures, it is crucial that researchers recognize that each ethical dilemma encountered during fieldwork is unique and rooted in social contexts, interpersonal relationships, and personal narratives.

## Background

Despite recent developments aimed at creating international guidelines for ethical global health research, critical disconnections remain between how global health research is carried out in the field and the institutional ethics frameworks intended to guide research practice. Researchers doing fieldwork face significant challenges in mediating between the ethical priorities and commitments of research institutions and the day-to-day concerns of low-income communities involved in the research.

Given that most global health research is conducted in the Global South with an aim to support “the study of health issues related to the low and middle-income countries of the world” [1], concerns about ethical research become even more important when considering multiple differences between North and South in relation to culture, power, inequalities, politics, and geographies [2, 3]. This is further complicated by recent changes over the past 15 years, in which funding calls for global health research projects now revolve around providing solutions or potential interventions to health problems that can be evaluated, measured, and eventually scaled up [4, 5]. New international collaborations dedicated to an implementation and evaluation research agenda have led to an increase in interdisciplinary and multinational global health projects that involve investigators or trainees from high-income nations participating in studies in LMICs [6, 7]. Numerous ethical guidelines, such as the Nuremberg Code [8], the Belmont Report [9], the International Ethical Guidelines for Biomedical Research Involving Human Subjects issued by the Council for International Organizations of Medical Sciences [10], and the Declaration of Helsinki [11], have been developed to guide the regulation of study design, ethical review, and standards of care in international research, and together they form a cornerstone of ethical research practice [12]. Despite these guidelines, complex and problematic ethical issues continue to emerge from the interplay between global research protocols and the ways in which such research is manifested locally.

The challenges of conducting research in developing countries have been examined by bioethicists, practitioners, and social scientists alike, who are intent on the question of how to apply universal ethical principles to local contexts in meaningful and relevant ways [12–16]. Much of this attention on tailoring ethics to local contexts has centred on the research subject or setting, leaving the researcher’s role in the process and relationship with participants out of focus. Yet local tailoring of ethics is not just about how guidelines are adapted to specific research subjects or populations; local ethics concern ethical issues that arise in the day-to-day relations between participants and researchers and present researchers with “ethically important moments” to consider [17].

The social relations between researchers and study participants form the fabric in which dialogue, information sharing, and negotiations that are central to ethical practice take place, and at the same time, in which data are collected. Anthropologist Michael Lambek [18] calls for an ethics that is “relatively tacit, grounded in agreement rather than rule, in practice rather than knowledge or belief, and happening without calling undue attention to itself” (p. 2). Indeed, beyond the direct effects of a research study or planned intervention that aims to manipulate dependent variables to influence behavioural, personal, organizational or political change, social relationships themselves can be seen as “unregarded interventions” in which the researcher’s presence and relationship with participants can affect the course and outcome of the study [19].

In this paper, we attempt to map out the ethical tensions likely to arise in global health fieldwork as researchers negotiate the challenges of balancing ethics committees’ rules and bureaucracies with actual fieldwork processes in local contexts. Drawing from our research experiences in Jamaica, we argue that ethical research is produced through researchers’ practices of reflexivity and negotiation of the intersubjective space created in the relationship between researcher and study participant. It also calls for researchers’ vigilance regarding issues of positionality and power that may arise at multiple levels [20]. In doing so, we position ethical research practice in global health as a dialectical movement between the spoken and unspoken or, more generally, between operationalized rules and embodied interpersonal understandings (or misunderstandings).

## Main text

### Dream-A-world program implementation and evaluation in Kingston, Jamaica

In this paper, we draw on experiences from a multi-partnered, interdisciplinary implementation and evaluation study conducted with primary school children in impoverished inner-city communities of Kingston, Jamaica. Our study explored the impact of a 2.5-year multifaceted mental health school-based intervention, called Dream-A-World (DAW), that was aimed at children between 8 and 10 years of age who were deemed by teachers to be at high risk of developing psychological and behavioural problems in the future. The intervention focused on school-aged children in severely disadvantaged inner-city areas of Kingston, Jamaica, with high rates of school dropout, crime, early pregnancy, poverty, unemployment, and gang violence documented as significant longstanding social stressors [21]. The overall goal of the DAW program was to reduce violence and promote resilience in children by preventing school failure or dropout and promoting self-control through pro-social culturally informed group activity [22].

The DAW study was designed by researchers from the Caribbean Institute of Mental Health and Substance Abuse (CARIMENSA), University of West Indies, and McGill University in Montreal, Canada, and received funding from Grand Challenges Canada to be conducted as a transition-to-scale study of the intervention. The DAW research team was subdivided into two teams: program implementation and program evaluation. The implementation team was comprised of art and cultural therapists distributed among the four schools, who worked alongside the academic teachers. The program implementation was supervised by two Jamaican psychiatrists, who were the principal investigators (PIs) of the study. The program evaluation team consisted of the four study PIs (two Jamaican psychiatrists; one Canadian psychiatrist, and one Canadian health scientist), three Jamaican psychologists, four Jamaican research assistants, and two social scientists (one Jamaican master’s student, one Canadian doctoral student).

#### DAW intervention implementation

The intervention, which took place between 2013 and 2015, was conducted in four schools in four resource-limited, inner-city communities, and was implemented as a case-controlled, non-blind, randomized trail. It involved more than 180 students, eight teachers, and an intervention implementation team of 16 therapists (including dancers, visual artists, musicians, and psychologists).

Forty to 50 students were selected from each school, for a total of 180 students taking part in the overall study. Children in the DAW intervention group attended a 3-week cultural therapy program that was held for two consecutive summers, beginning at the end of the children’s grade 3 school year and continuing until the start of their grade 6 school year. The summer program was divided into two components. In the morning session, the children received remedial classes in language and mathematics delivered by teachers from their schools who were participating in the program. In the afternoon session, the children took part in the Dream-A-World process, which invited them to imagine their life on a new planet with whatever social and ecological settings and animals they wished to have. During this time, the cultural and art therapy team worked with the children to construct songs, poems, music, and dances about their worlds with the aim of putting together a dramatic performance that was presented to parents, teachers, and the community on the final day of the program. Social skills, self-control, and agency were said to be instilled through this creative process, promoting resilience, interpersonal communication, and the development of self-esteem [23]. The students participating in the DAW study also received a meal during each program session. Following the 3-week summer workshop, the students received bi-monthly top-up sessions during the school year. During this time, the art and culture implementation team continued to work with the children after school.

#### DAW program evaluation

The evaluation of the DAW implementation was undertaken by a team of mental health practitioners and social scientists and was intended to demonstrate the impact of the intervention and assess the potential efficacy of its being scaled up to other schools across the island. Study activities included international university collaboration, training child mental health and education staff, and strengthening school and family partnerships as part of task-shifting strategies to build infrastructure aimed at child and youth mental health. Depending on the outcomes of the transition-to-scale study, the Jamaican Ministry of Education was considering expanding the program to another 1000 schools across the country.

To assess the effectiveness of the program, the DAW evaluation team used a mixed methods approach to evaluation that included a matched comparison group design, as well as an ethnographic case study of one of the schools. Table 1 summarizes the overall design of the evaluation study. Specifically, fieldwork was conducted over the course of three years and included: (a) hiring and training of implementation and research team members; (b) sociodemographic surveys with over 180 students and collection of secondary data sources (e.g. report cards) from the teachers; (c) pre- and post-program psychological assessments for each of the students in the intervention and control groups; (d) in-depth interviews and focus groups with students, parents, teachers, program facilitators, and other stakeholders involved in the intervention to explore their perspectives on the effectiveness of the DAW intervention; (e) an ethnographic study in one of the four schools to help the research team gain a deeper understanding of the larger social, economic, and political factors governing the children’s lives; and (f) data entry, interview transcription, and analysis of both qualitative and quantitative data.

**Table 1:** DAW Implementation and Evaluation Process

Phase 1 (February–May 2013)	The research team consulted with primary school teachers in the four schools to select the students who would participate in the DAW program (intervention and control cohorts). Research assistants administered sociodemographic surveys among all participating students from both cohorts in the four schools. Secondary academic data (e.g. report cards and teacher assessments) were collected for each student, and pre-study psychological testing was done to determine baseline scores before program implementation. Psychological instruments used were: ASEBA (Achenbach System of Empirically Based Assessment); Child Behavior Checklist (CBCL – Parent and Teacher); Wechsler Intelligence Scale for Children, Fourth Edition (WISC-IV); Wide Range Achievement Test, Third Edition (WRAT-III); Mico Diagnostic Reading Test (MDRT); and the Piers-Harris Children’s Self-Concept Scale, Second Edition. During this phase, three full-day training sessions on how to implement the DAW intervention were conducted with the cultural therapy team (dancers, musicians, psychologists, visual artists) by the study PIs.
Phase 2 (June 2013–July 2014)	The DAW 3-week summer intervention sessions were conducted in the summer months (Summer 2013 and Summer 2014). In Summer 2013, focus groups and in-depth interviews were conducted with teachers and parents to determine the types of issues facing students in the community. Focus groups were also conducted with the DAW program cultural therapy team to better understand their experiences with the process of implementing the DAW program.
Phase 3 (July 2014–August 2015)	In Summer 2014, post-program interviews were conducted with teachers and parents to understand how the DAW program had affected the children who participated in the intervention. Research assistants also collected secondary academic data (e.g. report cards, teachers assessments) on all the students who participated in the DAW intervention (both intervention and control cohorts), and post-program psychological testing was conducted to determine the effects of the intervention. An in-depth ethnographic case study was rolled out in one of the four schools of the DAW program to gain a deeper understanding of the larger social, economic, and political factors governing the lives of children living in communities affected by high rates of violence and economic deprivation. Ethnographic data collection methods included participant observations, in-depth interviews with parents, teachers, guidance counsellors, and community members, as well as small group discussions with the students (*n* = 28) who took part in the DAW program in that school.
Phase 4 (July 2014–August 2016)	Data entry, analysis, and transcription were performed by the research assistants, social scientists, and study PIs. Data from the ethnographic research were analyzed and results shared with the children participating in the sub-study.

### Negotiating a relational ethics in global health fieldwork

In any type of field research, researchers insert themselves into the everyday aspects of their study participants’ lives. The intersubjective relationships they develop with participants are not, of course, limited to the specificity of the study or intervention and inevitably spill over into the outside, everyday world. The formation of relationships inevitably places issues of power and positionality at the centre of the fieldwork process, and these issues are further complicated in research contexts, where unequal power structures underlie the collaborations taking place. This is especially true for research aimed at tackling inequalities in socioeconomically breeched settings, as well as research in contexts where a legacy of colonialism, combined with gender-biased or patriarchal systems, reinforces general cultural notions of power and control and challenges any epistemological or methodological approaches that seek to include the voices of usually marginalized participants. Given the nature of these intersubjective relationships, ethics in global health research should not only be “concerned with regulatory ‘protection’ of subjects but [also] with ‘taking care’ of and with relations with multiple others” [7] (p. 30). It is not only intersubjective relationships between researchers and participants that affect the research process; “researcher identity” can affect global health research in complex ways. Simon and Mosavel [24] postulate “that all global health research has a visible ‘face’, be it that of a foreign investigator, a local scientist and collaborator or a hired staff member. This face comes to represent a given research project by way of a subject’s associations, claims to power and knowledge and ascriptions of race, gender and culture” (p. 84). In the present study, the combination of highly charged topics, in-depth and long-term contact with research participants, and the evolving emotional environment of the researchers’ own social worlds required navigating some ethical uncertainties of the researcher–participant relationship. In the section below, we outline three aspects of the research process that presented researchers with ethical challenges in conducting global health research.

#### Informed consent and the conflict of obligation

Informed consent is one of the central regulatory norms that all research ethics review boards or committees demand researchers respect and document [25, 26]. All research projects are required to go through a process of procedural ethics that involves preparing documents of *informed consent* and *assent* (in the case of studies involving children). Informed consent has become so tied with and embedded into the research process that research protocol approval, funding, and publication of study results are all dependent on whether an appropriate consent process was followed [8]. In health research involving human subjects, consent to a therapy or a research protocol must possess three features to be valid: it must be given voluntarily, it must be expressed by a research subject who is competent and autonomous, and the subject must be adequately informed [27]. Beyond these features, the Canadian Tri-Council Policy Statement on ethics additionally stipulates the need for consent to be an ongoing process throughout the research project [28]. Participants must be informed about their rights and the procedures for taking part in the research, including the harms and benefits associated with participating, how the collected data will be protected and used, and their rights to discontinue participation or withdraw from the study at any time [11, 27]. It is only once potential participants fully understand the scope and purpose of the research that they are considered able to make an “informed” decision about whether to participate.

Yet ideas of who is considered competent and autonomous vary from context to context, and especially in global health projects engaging with children, who are often perceived as vulnerable, dependent, and needing protection [29]. However, in recent years it has been well established that, in research conducted with children, as asserted by the UN Convention for the Rights of the Child (1989), children have the right to express their views on all matters that affect them and to seek, receive, and impart information and ideas. Fundamental to these rights is the child’s ability to make sense of and understand the information he or she receives in order to be able to consent and make an informed decision about taking part in the research. In recent years, researchers have put critical thought into ensuring that the process of documenting informed consent does not inadvertently disenfranchise people whose voices need to be heard. In light of this, researchers have engaged in alternative means of acquiring and recording consent, through community engagement, verbal consent techniques, and art-based methods, to ensure the process is culturally sensitive to different population needs [30, 31].

In the DAW study, informed consent was first sought from research participants before they engaged in the program and then again throughout the course of the study, as well as during evaluation and ethnographic fieldwork. To uphold respect for a child participant’s dignity, the child’s assent or willingness to participate was sought in addition to the parents’ informed consent. This was done by explaining to the child the purpose of the research project and the child’s role in the process. Even though participants provided consent to participate, it can be assumed that what constituted the ability to provide valid informed consent could have been influenced by the social construction of the research context. The problematic aspect of the consent process is that once consent is culturally validated and adequately explained, research participants are considered to have made “informed” choices to “voluntarily” participate in research—an assumption that is, at minimum, questionable. While the normative assumptions of the consent process are able to uphold the rights of the research subject, they can concurrently ignore key insights into the structural conditions faced by research participants—including poverty, access to health care, and political insecurity [25, 26]. In many ways, since informed consent procedures are standardized, simple, and easily compatible with differing research contexts, they function to quell the moral concerns of the researcher, academic and funding institutions, yet can also take on the form of an “empty signifier”, an image onto which people project their intentions and hopes, but which in practice remains unaffected [32]. This is especially true for health research being conducted in LMIC contexts, where the procedural consent process can often produce a form of active “unknowing” in the researcher. According to Geissler [7], “unknown knowns” refer to the researcher’s being aware of certain structural conditions that underlie socioeconomic inequalities while deciding to remain silent as a means to achieve scientific production.

In the present study, the inseparability of the intervention’s implementation from the research aspects of the study complicated the consent process considerably. Ethical issues of seeking informed consent in this field context were entangled in complex circumstances, in that, because the DAW team was providing an intervention in the community, people’s willingness to participate may have been influenced by explicit or tacit expectations of assistance. Given the desperate living conditions of people in the neighbourhood, parents and community members recruited to be interviewed about life in the inner-city expressed disbelief that researchers working closely with a Canadian university and the Jamaican Ministry of Education would come to ask children and families questions regarding their lives, but could not subsequently provide assistance with school-related expenses or allowances to support their participation in the DAW intervention. Many parents assumed the researchers would take care of the children and families after finding out more about their socioeconomic situations and lived experiences. Even after it was explained to them that the purpose of the study was only to create knowledge related to the research topic and that they would receive no direct material or economic benefits for participating in the study, many parents, especially those whose children were not part of the program implementation trial, were adamant to have their children participate in the research. In most cases, it was not easy to ascertain whether the children and families accepted to participate because they fully understood the consequence of participating, or whether they were hoping they would eventually get a place within the DAW program. This is a well-documented phenomenon in health-related research in low-income countries, where poverty may be a driving force for research participation for people with few other options for accessing health resources or forms of care [7, 33–36].

The consent scenario raises concerns around doing extended research on vulnerable populations and the possibility that researchers might inadvertently cause harm through ongoing encounters and questions. How can this “unknown known” be actively exposed, acknowledged and negotiated? How can we find opportunities in a study to unpack and reflect on the ethical tensions surrounding the process of consent? This calls for a reflexive approach in which researchers are encouraged to acknowledge both spoken and unspoken gestures during the research process [37] as well their own positionalities and identities. One of the main discrepancies between formal ethics and local perceptions concerns differentials of power and wealth within a community, which formal protocols usually do not address. The cultural therapy and research teams of the DAW study were composed of university graduates from middle-class communities in Kingston, whose socioeconomic circumstances and political histories were very different from those of the study participants of the DAW program. The research partners from Canada introduced further layers of difference in terms of culture, race, class, socioeconomic status, and education. Most formal ethics guidelines place such political-economic differences outside the professional responsibility of science as a means of remaining detached from the world.

In global health research, the expectations regarding what constitutes adequate consent can vary depending on multiple factors, such as who is interacting with whom, levels of engagement between researchers and subjects (or other actors), and how definitions of consent are operationalized. While researchers may be required to meet the demands of securing informed consent as defined by external agencies or individuals, consent should be thought of as an ongoing dialogue between researchers and participants that requires constant negotiation and renegotiation. Reubi [38] suggests that informed consent does not simply allow individuals to make a choice, it also produces particular subject positions and narratives about what being a person means.

#### Confidentiality and anonymity of participation

Maintaining anonymity and confidentiality is considered a central tenet for the conduct of ethical research [39]. Conventional quantitative data collection methods, such as questionnaires, assessments, and observations, make it relatively easy to protect a participant’s anonymity and confidentiality, usually by assigning identification numbers. In qualitative research, researchers may use several strategies for maintaining confidentiality with participants, such as assigning pseudonyms when reporting data, or leaving out details of participants (e.g. traits, characteristics) that could be easily identified [40]. During the DAW data collection process, as in many other health studies, the research team assured participants that only members of their team would have direct access to records of the data collected about them and that their actions or words would not be disclosed to others in the field, such as family members, teachers, or other community members. A significant portion of the research time was spent discussing the use of pseudonyms with participants and encouraging them to choose fictitious names for themselves, their friends, and family members. Although this is a long-established convention, some of the contradictions encountered most frequently in our study had to do with important discrepancies between notions of confidentiality expressed within an institutional ethics framework, such as an IRB review, and the concerns voiced by research participants in the fieldwork setting.

For example, the children involved in the study sometimes challenged this issue of anonymity and confidentiality, such as when they elected to report their own results or wanted their contributions to be acknowledged. An example of this occurred at the end of the second summer session, when the children in the intervention group were performing a play for the community and were required to wear masks to protect their identities from the audience, as they were being video-recorded for education purposes (to be used in the training manual for the DAW program). While the distribution of children into intervention and control groups was not a blinded process—meaning that the children, caregivers, and teachers knew to which groups the children were assigned—the research team wanted to ensure the children’s identities remained confidential from the wider community. However, during their performance, many children began removing their masks and wanted to reveal their faces, precluding any chance of protecting their identity. The issue of anonymity is especially salient in research that is engaged in “transformative action”, in which the research is deemed to give “voice” to individuals who have historically been silenced by oppressive power structures. For the children on stage, removing their masks was a gesture to celebrate their personal stories of growth and resilience with their families and community. The intervention team was witness to their growth over the course of the two years of the program, and the children wanted to share their stories and accomplishments with the individuals who had been part of the DAW process and with their families; they also wanted the team to use their identities in reporting the data.

It has been suggested that researchers can achieve more authentic ethical engagement by enabling participants to explore their choices and make decisions about concerns related to confidentiality, rather than the researchers making all the decisions. In the case of the DAW project, the children were given an opportunity to discuss with the researchers the importance of showing their school and community what they had achieved by participating in the DAW process. The team also engaged the children in discussions of why the researchers were trying to protect their confidentiality. These back-and-forth discussions resulted in a negotiation of the children’s rights as study participants, where together it was decided that certain outcomes of the DAW project (e.g. drawings and art work) could be shared with family members and friends but would remain anonymized for publication in other venues. These types of discussions made the research experience meaningful for participants, but also allowed the research team to create more balanced power relations that allowed more respectful relationships to be built between researchers and participants.

#### Researcher–participant relationships in an era of global communication

In field research, the researcher can be considered to be the research instrument, and as such, the data collected are intersubjectively constructed and embedded between researcher and participant. The mere presence of the researcher may affect events and outcomes of the study [41]. In the conduct of sensitive research, researchers often grow close to participants, which can blur the line between the roles of friend and research subject [42]. Researchers engaged in field research do not simply neutrally observe and objectively collect data with scientific detachment. These difficulties associated with the blurring of the line between research and friendship have been articulated by a number of authors [43–45], and the term “researcher-friend” [46] has been used to acknowledge that researchers often get involved in friendship-like relationships with research participants. In fact, to a large extent the data collection process is dependent on an optimal researcher-friend relationship that cannot be standardized. This raises ethical problems if researchers collect data that participants did not plan on sharing with them but divulged based on a perception of closeness. This type of relationship can also raise issues when the research relationship is forged with individuals from vulnerable groups, such as children.

Over the course of the DAW study, the more the research team associated with the parents and children informally (e.g. hanging out in the school courtyard during lunch, emailing, texting, phone calls), the more familiar the study participants became with the research team, and the roles of researchers began to blend with other roles in the field. The longitudinal nature of the study allowed the research team to build rapport with the study participants over time. The ability to build rapport and trust between researchers and participants was vital to unearthing “subjugated knowledge” [47], the lived experiences of research participants and the meanings they ascribed to those experiences. Yet rapport was built differentially between researchers and youth study participants depending on the experimental groups to which the children were assigned. Children in the intervention cohort were in contact with the cultural therapists and research team members more often than were those assigned to the control cohort. Some researchers were even adopted into fictive kinship networks of the children, as formal greetings of “Miss” or “Mr.” evolved into greetings of “Aunty” and “Uncle” when meeting with the researchers. As emotional bonds grew among researchers and research participants, interpersonal relationships became more layered and complex. Many researchers in the DAW study wondered how they would feel when the research was over and what difficulties they might encounter in ending their relationship with the children.

Upon completion of the study, a number of researchers maintained some level of contact with the children and other participants over a short period of time, as a means of bringing closure to the relationships they had developed and of keeping track of the participants for eventual follow-up. The amount of contact differed considerably among researchers in the project, from some having minimal contact with the children (e.g. assessment psychologists), to others maintaining extended contact with them for a number of months after the project’s completion (e.g. social scientists). While termination of the research relationship was managed via an exit strategy in place within the protocol, managing the social relationships created in the research process proved more difficult. Leaving the fieldsite did not necessarily guarantee a distancing between researcher and participant. Study participants were able to reconnect with some of the researchers via social media platforms such as WhatsApp and Facebook based on the contact information found in the consent forms (e.g. phone number and email address). This ongoing nature of the relationship took some of the researchers by surprise, as they did not have strategies in place to deal with this. These ethical dilemmas prove that ethical practices are not easily safeguarded by formal ethical precepts alone and need to be produced in social relations between researchers and study participants [48]. Relational boundaries between researcher and participant must be negotiated and renegotiated as an ongoing part of the research process, in an effort to achieve a balance in dealing with issues of power, access, and respect in the research relationship. Research teams must also reflect sufficiently on these relational boundaries throughout the research process to think consciously and critically of how these interrelational factors might shape the project’s results and outcomes, especially in implementation, evaluation, and scale-up studies.

## Conclusion

There are significant challenges in carrying out interdisciplinary multimodal health research among vulnerable populations in LMIC settings where multiple forms of adversity co-exist. In addition to the typical issues raised in the implementation of international health studies, the ethical challenges outlined above call us to broaden our definition of ethics and rethink our measures of ethical research conduct in ways that are more flexible and reflexive. Research ethics in global health should not be based solely upon passive acceptance of existing guidelines on ethical conduct, but on tacit modes of knowing that are responsive to and engaged with research participants. Research teams can conduct research projects ethically by ensuring a practice of joint reflexivity between researchers and participants that stresses transparency and accountability in the study design and throughout the research process. A joint reflexive relationship is also fostered by paying careful attention to the ways in which issues of power, identity, and positionality are acknowledged and managed in the project [17]. In a reflexive relationship with study participants, researchers engage in an “ethics-of-care” approach that recognizes that ethical decision-making between researcher and participant has both a cognitive and an emotional component [49]. Relationships in the field are affected not only by the content of the research project, but also by the social, economic, and political context in which the study is carried out and by researchers’ and participants’ personal motivations. Rather than trying to operationalize ethical practice through a list of forms and procedures, researchers must recognize that each ethical dilemma encountered during fieldwork is unique and can be seen as rooted and embedded in social contexts, interpersonal relationships, and personal narratives. In carrying out global health implementation and evaluation research in LMIC settings, the process of joint reflexivity between researcher and participant is important for creating a space to generate ethical and moral maps by which research terrains can be navigated in more appropriate, conscious, and meaningful ways.

## Abbreviations

ASEBA: Achenbach System of Empirically Based Assessment
CARIMENSA: Caribbean Institute of Mental Health and Substance Abuse
CBCL: Child Behavior Checklist
DAW: Dream-A-World
IRB: Institutional Review Board
LMIC: Low-and-middle income country
MDRT: Mico Diagnostic Reading Test
PI: Principle investigator
UN: United Nations
WISC: Wechsler Intelligence Scale for Children
WRAT: Wide Range Achievement Test
